# Emerging links between TLR4 signaling and ferroptosis in heart failure

**DOI:** 10.3389/fphar.2026.1909522

**Published:** 2026-10-01

**Authors:** Ci Wang, Xiangyuan Huang, Zeyu Zhang, Ying Hu, Dongming Lin, Shuwei Huang

**Affiliations:** 1 The First Affiliated Hospital of Zhejiang Chinese Medical University (Zhejiang Provincial Hospital of Chinese Medicine), Hangzhou, China; 2 School of Traditional Chinese Medicine, Zhejiang Chinese Medical University, Hangzhou, China; 3 Graduate School, Beijing University of Chinese Medicine, Beijing, China; 4 First Teaching Hospital of Tianjin University of Traditional Chinese Medicine, National Clinical Research Center of Chinese Medicine Acupuncture and Moxibustion, Tianjin, China; 5 Graduate School, Tianjin University of Traditional Chinese Medicine, Tianjin, China

**Keywords:** ferroptosis in cardiomyocytes, heart failure, iron metabolism, lipid peroxidation, TLR4 signaling pathway

## Abstract

Iron is an essential nutrient for living organisms, and the maintenance of its homeostatic balance within the body is a critical factor in ensuring normal physiological functions. Cardiomyocyte ferroptosis has emerged as a potential contributor to heart failure progression, involving complex regulatory mechanisms that remain incompletely understood. While the TLR4 signaling pathway was traditionally regarded as a therapeutic target for chronic inflammatory conditions, emerging evidence indicates that its activation may contribute to ferroptosis in cardiomyocytes. The proposed mechanisms through which TLR4 signaling may regulate cardiomyocyte ferroptosis are systematically summarized, and several therapeutic strategies targeting TLR4-associated ferroptosis are evaluated. Furthermore, promising avenues for future investigation are discussed, with particular emphasis on the need for direct mechanistic validation in cardiomyocytes and *in vivo* models, which may ultimately inform novel therapeutic strategies for heart failure.

## 1 Introduction

Heart failure (HF) is a leading cause of death and disability in China and worldwide (Zhou et al., 2016; Global Regional, 2015). According to the American Heart Association, heart failure was mentioned on approximately 300,000 death certificates in the United States in 2013, with total direct and indirect costs of all heart diseases estimated at $207.3 billion (Mozaffarian et al., 2016). More recent projections indicate that the prevalence of HF is escalating rapidly, with an expected increase of 34% in the coming decades. In the United States, total direct medical costs for HF are forecast to rise from $21 billion in $2012 to $53 billion by 2030, and total costs, including indirect expenditures, are estimated to grow from $30.7 billion to $69.8 billion over the same period (Maddox et al., 2024). In the Olmsted County Cohort Study, the 1-year and 5-year mortality rates for all types of HF patients diagnosed between 2000 and 2010 were 20% and 53%, respectively. A combined analysis of the Framingham Heart Study and the Cardiovascular Health Study reported a 5-year mortality rate of 67% among HF patients after diagnosis (McDonagh et al., 2021). HF severely impacts patients’ quality of life and lifespan, imposing a heavy economic burden on families and society. To date, there is no definitive cure for HF, making its prevention and treatment a pressing global health challenge.

In 2019, Fang et al. (2019) first proposed that ferroptosis represents a key novel mechanism in cardiac diseases. The study systematically suggests that ferroptosis not only occurs in chemotherapy-induced and ischemia-reperfusion-induced cardiac diseases but also that targeted intervention against ferroptosis may alleviate disease progression in these models. Subsequent studies have further expanded this concept, demonstrating that ferroptosis also plays a critical role in post-myocardial infarction heart failure and can be effectively suppressed by activating protective signaling axes such as the cGMP-PKG-GPX4 pathway, which restores redox homeostasis and limits oxidative stress-mediated myocardial injury (Zhang et al., 2026). Therefore, inhibiting ferroptosis in cardiomyocytes is a critical strategy for protecting cardiac function and preventing HF. The regulatory pathways of cardiomyocyte ferroptosis are of significant research value and have considerable potential for clinical translation. Previously, the TLR4 signaling pathway was primarily recognized as a key regulator of inflammatory responses, present in tissues such as cardiomyocytes (Zhang et al., 2024) and blood cells (Wen et al., 2021; Vallance et al., 2017). Research by Li et al. (2019) suggested that activation of the TLR4 signaling pathway may be associated with ferroptotic changes in cardiomyocytes, although the underlying regulatory network remains incompletely understood. This review systematically summarizes recent advances in the potential mechanisms linking TLR4 signaling and cardiomyocyte ferroptosis, aiming to outline its potential underlying mechanisms. Additionally, it briefly introduces several effective drugs targeting TLR4 to prevent and control ferroptosis, followed by a discussion that provides insights and direction for future research. The goal is to provide insights into potential mechanisms and therapeutic directions for HF prevention and management.

## 2 Mechanisms of ferroptosis

Ferroptosis is a form of regulated cell death involving the coordinated interplay of multiple pathways, including dysfunction of the cystine/glutamate transport system, iron metabolism dysregulation, and lipid peroxide accumulation. These pathological processes intertwine, leading to excessive intracellular lipid peroxide production that exceeds the cell’s antioxidant capacity. When the accumulation of these peroxides exceeds the cellular antioxidant capacity, ferroptotic cell death may occur (Figure 1).

**FIGURE 1:**
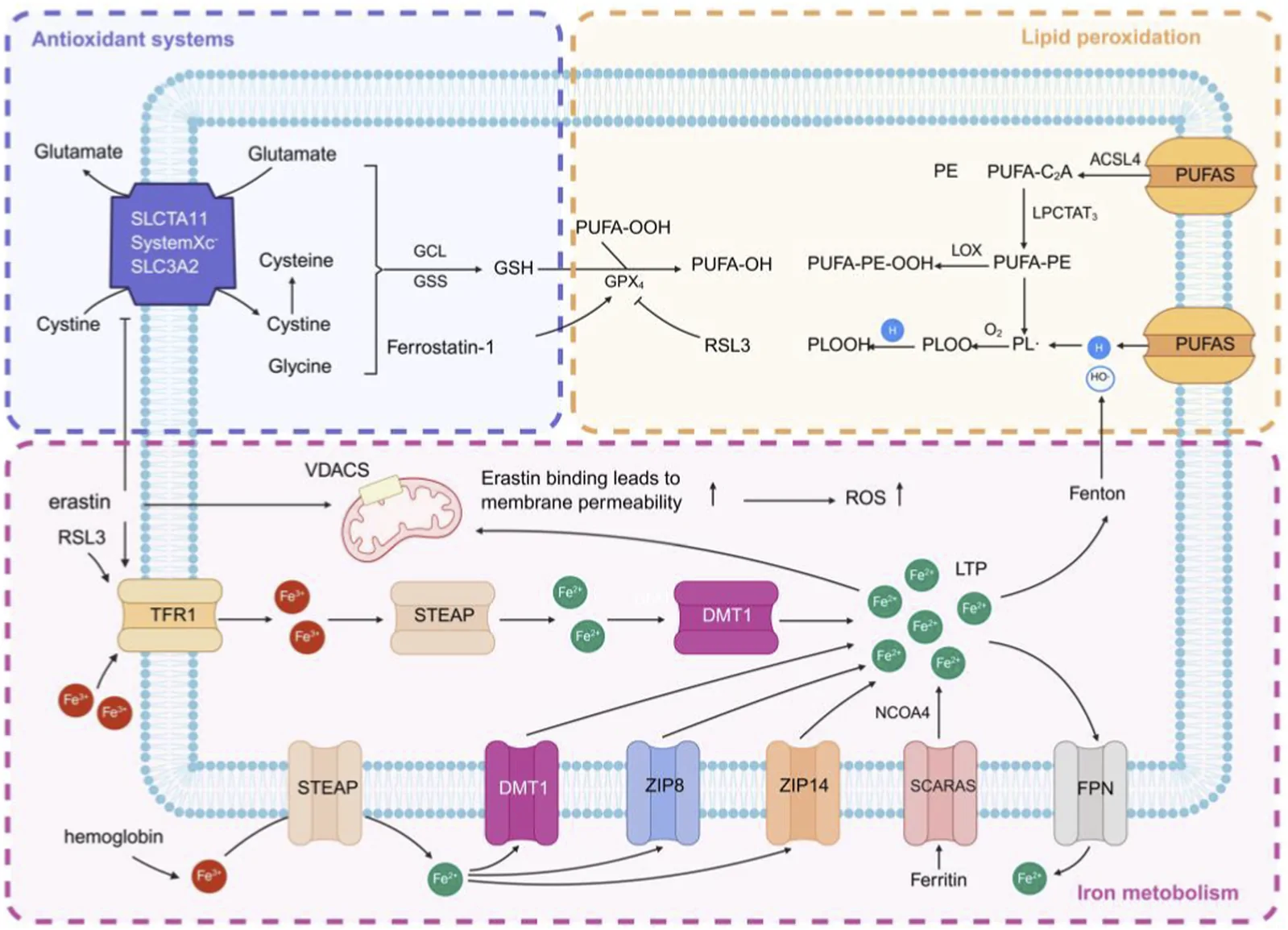
Impaired cystine/glutamate transport, abnormal iron metabolism, and lipid peroxidation during ferroptosis. Excess Fe^2+^ enters the unstable intracellular iron pool via multiple pathways, where it reacts with H_2_O_2_ in a Fenton reaction to generate hydroxyl radicals (HO•). HO• oxidizes the polyunsaturated fatty acyl groups of phospholipids (PUFA-PL) to produce phospholipid hydroxyl radicals (PLOOH). During this process, PUFA-PL donates hydrogen atoms to HO•, forming PL•. Subsequently, PL• reacts with O_2_ to generate phospholipid hydroperoxide radicals (PLOO•). PLOO• then acquires hydrogen atoms from another PUFA-PL to form PLOOH. In the presence of Fe^2+^, PLOOH cleaves to form phospholipid alkoxy radicals (PLO•). These radicals acquire hydrogen atoms from another PUFA-PL to generate phospholipid alcohol (PLOH) and new PL•, initiating another lipid radical chain reaction. Ultimately, PLOOH decomposes into 4-HNE and MDA, triggering ferroptosis. Additionally, LOX, POR, and COX2 directly catalyze the oxidation of PUFA-PL to produce PLOOH. GPX4 converts toxic PLOOH (PUFA-OOH) into the corresponding alcohol PLOH (PUFA-OH), effectively preventing intracellular lipid peroxide accumulation. GSH is a crucial factor in maintaining GPX4’s antioxidant activity. When System xc^−^ transport is inhibited, intracellular cystine deficiency prevents sustained GSH synthesis. This not only limits the cell’s radical scavenging capacity but also inactivates GPX4. Excessive accumulation of ROS and lipid peroxides within the cell ultimately leads to cell death. Created in BioRender. Huang, X. (2026) https://BioRender.com/7xxveuj.

### 2.1 Cystine/glutamate transport impairment

The cystine/glutamate antiporter (System xc^−^) is a transmembrane amino acid transporter located on the mammalian cell membrane. It consists of two subunits: a light chain (solute carrier family 7 member 11, SLC7A11) and a heavy chain (solute carrier family 3 member 2, SLC3A2). Its activity is primarily regulated by SLC7A11 (Bridges et al., 2012). Under normal metabolic conditions, extracellular cystine is transported into cells via System xc^−^, while System xc^−^ simultaneously transports intracellular glutamate out of cells in an equivalent ratio. This antiport system maintains amino acid equilibrium between intracellular and extracellular spaces (Bi et al., 2019; Liu et al., 2019). Cystine entering the cell is further reduced, breaking its disulfide bond to form cysteine. This essential precursor subsequently combines with intracellular glutamate and glycine to form glutathione (GSH). GSH serves as a crucial broad-spectrum antioxidant and reactive oxygen species (ROS) scavenger within mammalian cells, efficiently scavenging various intracellular free radicals to maintain cellular redox homeostasis (Wan et al., 2019). Glutathione peroxidase 4 (GPX4) catalyzes the reduction of phospholipid hydroperoxides using GSH as an electron donor, producing glutathione disulfide (GSSG). Concurrently, GPX4 converts toxic PUFA-OOH into non-toxic PUFA-OH, effectively preventing the accumulation of lipid peroxides within cells (Latunde-Dada, 2017). GSH is a crucial factor in maintaining GPX4’s antioxidant activity, and together they form a vital antioxidant system within mammalian cells. When the transport function of System xc^−^ is inhibited, cells lack cystine and cannot sustain GSH synthesis. This limits the cell’s ability to scavenge free radicals and simultaneously inactivates GPX4. The resulting accumulation of ROS and lipid peroxides promotes ferroptotic cell death (Wang L. et al., 2020). Erastin, one of the earliest identified ferroptosis inducers, primarily inhibits System xc^−^ activity, thereby blocking cystine uptake. Reduced intracellular cystine availability limits cysteine production and GSH synthesis, ultimately promoting ferroptosis (Dolma et al., 2003). Subsequently identified agents such as sulfasalazine, glutamate, and sorafenib exhibit similar effects (Sehm et al., 2016; Lachaier et al., 2014). Erastin also affects mitochondrial function by interacting with voltage-dependent anion channels (VDACs), leading to increased oxidative stress and contributing to ferroptotic cell death. This leads to the release of intracellular oxidants, ultimately causing cell death (Skonieczna et al., 2017). RSL3 primarily targets GPX4 inhibition to induce ferroptosis (Cheff et al., 2023), while Ferrostatin-1 suppresses ferroptosis primarily by acting as a radical-trapping antioxidant that interrupts lipid peroxidation chain reactions (Wang et al., 2023).

### 2.2 Iron metabolism disorder

Iron is an essential trace element in the human body and plays indispensable roles in various physiological processes. It performs vital biological functions within the body, such as catalyzing various biochemical reactions by accepting and donating electrons, forming hemoglobin to transport oxygen, participating in mitochondrial energy release (Camarena et al., 2021). Deficiency of iron in the human body can lead to a series of clinical syndromes characterized primarily by anemia and abnormalities in iron-dependent enzymes manifesting as hemolytic anemia, jaundice, pica, spoon-shaped nails, etc (Pasricha et al., 2021). Iron within the body is divided into functional iron and storage iron. “Functional iron” accounts for approximately 75% of total iron content, primarily manifesting as hemoglobin iron, myoglobin iron, and transferrin iron, responsible for oxygen transport. Small amounts of iron are also present in enzymes such as cytochrome P450, cytochrome oxidase, peroxidase, and catalase, participating in biological oxidation processes. Additionally, approximately 25% of iron is stored in the liver, spleen, and bone marrow in the form of ferritin and hemosiderin, serving to replenish functional iron when needed (Saint-Germain et al., 2017; Hassan et al., 2016). In healthy individuals, the iron required for daily hematopoiesis primarily originates from aged and destroyed erythrocytes. However, the body must also maintain iron metabolism balance by acquiring iron from dietary sources (Dutt et al., 2022).

The mechanisms of cellular iron uptake primarily involve four pathways, among which the transferrin-mediated uptake pathway involving transferrin receptor 1 (TFR1) (Nemeth and Ganz, 2023) is the most critical: trivalent iron from dietary sources is reduced to divalent iron in the acidic gastric environment. After intestinal absorption, ceruloplasmin re-oxidizes it back to trivalent iron, which then binds to transferrin for transport via the bloodstream to tissue cells (Piskin et al., 2022). This complex binds to TFR1 on the cell membrane surface, enters the cell via endocytosis, and is subsequently reduced to ferrous iron by the metalloreductase STEAP3 within the endosome. Finally, with the assistance of SLC11A2 (DMT1), it is released into the cytoplasm (Kleven et al., 2015). Beyond this classical pathway, multiple iron reductases in serum (such as the STEAP family, iron chelator reductase 1, and cytochrome B reductase 1) can also reduce ferric iron to ferrous iron, which then enters cells via membrane transporters ZIP8, ZIP14, or DMT1 (Knutson, 2019). In monocytes and macrophages, a specialized mechanism exists for direct uptake of ferrous iron from the porphyrin ring of hemoglobin (Kristiansen et al., 2001). Furthermore, Li et al. (2009) demonstrated that ferritin can undergo endocytosis via the SCARA5 receptor, providing an additional supplementary pathway for cellular iron supply.

Intracellular Fe^2+^ participates in and regulates key biological processes through multiple pathways. A portion of Fe^2+^ is transported into mitochondria via mitoferrin-1 (MFRN1) and mitoferrin-2 (MFRN2), where it participates in cellular respiration and assists in the synthesis of Fe-S clusters and heme (Shaw et al., 2006). Another portion of Fe^2+^ is stored in the unstable iron pool, where iron ions exist in a dynamically adjustable state. This pool maintains cellular iron homeostasis and may contribute to ferroptosis under conditions of excessive iron accumulation (Brawley et al., 2023). Additionally, Fe^2+^ can bind to ferritin to form stable storage complexes (Zaugg et al., 2022). Under conditions of activated ferritinophagy, NCOA4 mediates ferritin degradation in lysosomes, releasing stored iron and increasing the labile iron pool (Quil et al., 2019); Another portion of ferritin is secreted extracellularly via the ferritin-export protein (prominin2) (Brown et al., 2019). Regarding iron efflux, ferroportin (FPN) is currently the only known cellular Fe^2+^ export protein, responsible for transporting a portion of Fe^2+^ back into the bloodstream to maintain overall iron homeostasis (Billesbølle et al., 2020).

Iron dysregulation is a major feature associated with ferroptosis. In 1894, British chemist Fenton discovered that hydrogen peroxide reacts with ferrous ions to generate hydroxyl radicals (HO•), a highly reactive oxygen species with potent oxidative properties that are harmful to the body (Zhao, 2023). Increased intracellular Fe^2+^ exacerbates the Fenton reaction and disrupts mitochondrial oxidative phosphorylation pathways, generating large quantities of highly cytotoxic hydroxyl radicals. When hydroxyl radical production exceeds cellular antioxidant capacity, they oxidize polyunsaturated fatty acids (PUFAs) on cell membranes into lipid peroxides (PUFA-OOH), disrupting membrane structural integrity. Simultaneously, they attack intracellular DNA and proteins, impairing cellular structure and function (Lei et al., 2022; Tang Z. et al., 2021). Studies indicate that stimulation of cells by different types of ferroptosis activators (such as erastin and RSL3) has been associated with increased TFR1 expression and reduced FTH1/FTL expression in certain models, potentially leading to enhanced iron uptake and decreased iron storage capacity (Bogdan et al., 2016; Yang and Stockwell, 2008). Geng et al. (2018) demonstrated that FPN knockout increased iron-dependent hydroxyl radical accumulation, thereby exacerbating erastin-induced ferroptosis in neuroblastoma cells. Furthermore, elevated autophagy also modulates iron overload-induced ferroptosis. For instance, Liu et al. (2023) demonstrated that TMEM164-mediated autophagy enhances iron accumulation and lipid peroxidation by degrading ferritin, GPX4, and lipid droplets; Rong et al. (2022) demonstrated that USP11 overexpression stabilizes Beclin 1, increases phagosome formation, and mediates ferroptosis; Yang et al. confirmed that SQSTM1 promotes ferroptosis by activating autophagy-dependent iron overload, exacerbating acute pancreatitis (Yang et al., 2023). Iron chelators such as deferoxamine reduce intracellular iron availability by binding free iron, whereas ferrostatin-1 and liproxstatin-1 inhibit ferroptosis as radical-trapping antioxidants that interrupt lipid peroxidation chain reactions (Kannan et al., 2023; Fernández-Acosta et al., 2022). Knocking out the TFR1 gene or upregulating ferritin in cells can also inhibit iron overload-mediated ferroptosis (Santagostino et al., 2021; Du et al., 2019). Therefore, regulating iron metabolism may become an important direction for modulating ferroptosis.

### 2.3 Lipid peroxidation

Intracellular accumulation of lipid peroxides results from ROS-mediated oxidation of biological membranes and represents a key molecular event associated with ferroptosis (Yang et al., 2016). Polyunsaturated fatty acids (PUFAs) are vital for maintaining the fluidity of cell membranes and organelle membranes, yet they are also the most sensitive lipids to lipid peroxidation. Among these, linoleic acid, arachidonic acid, and adrenic acid exhibit particularly high sensitivity to ferroptosis (Tang D. et al., 2021; Kagan et al., 2017). The lipid peroxidation process primarily involves two mechanisms: spontaneous oxidation and enzyme-catalyzed oxidation. Spontaneous oxidation is a radical chain reaction catalyzed by iron and oxygen. Under hydroxyl radical-mediated oxidation, PUFAs lose hydrogen atoms to form carbon-centered lipid radicals (L•), which rapidly react with oxygen molecules to generate lipid peroxy radicals (LOO•). LOO• further abstracts hydrogen from other PUFAs to form lipid hydroperoxides (LOOH). In the presence of Fe^2+^, LOOH can convert to lipid alkoxyl radicals (LO•). These newly generated radicals continue attacking adjacent PUFAs, thereby sustaining the chain reaction process. The enzymatic oxidation process relies on the synergistic action of multiple enzymes. Long-chain acyl-CoA synthase 4 (ACSL4) first catalyzes the conversion of polyunsaturated fatty acids into PUFA-CoA. Subsequently, under the catalysis of lysophosphatidylcholine acyltransferase 3 (LPCAT3), PUFA-CoA combines with phosphatidylethanolamine (PE) to form PUFA-PE. These PUFA-containing phospholipids serve as preferred substrates for oxidation and are ultimately converted into lipid peroxides under the catalysis of lipoxygenase (LOX), thereby completing the enzymatic lipid peroxidation process (Stockwell et al., 2017). Doll et al. (2017) knocked out ACSL family genes in mouse embryonic fibroblasts and found that only knocking out the ACSL4 gene significantly inhibited ferroptosis. Other enzymes besides ACSL4 did not induce ferroptosis, further supporting the essential role of ACSL4 in promoting ferroptosis through regulation of membrane phospholipid oxidation. Furthermore, Doll et al. (2019) identified FSP1 as a ferroptosis-suppressive factor, particularly effective in preventing polyunsaturated fatty acid peroxidation when co-administered with α-tocopherol and coenzyme Q10 (CoQ10). Thus, lipid peroxidation represents a central biochemical process underlying ferroptosis.

## 3 The TLR4 signaling pathway and ferroptosis

TLR4 signaling has been reported to regulate multiple ferroptosis-related molecules across diverse cell types, potentially influencing cardiomyocyte ferroptosis through direct or indirect mechanisms. Canonically, TLR4 activates both the MyD88-dependent and TRIF-dependent signaling pathways, although current evidence linking ferroptosis is predominantly derived from the MyD88-dependent cascade. The relevant mechanisms are summarized as follows (Figure 2). The TLR4 signaling pathway participates in the regulation of ferroptosis-related proteins through a multi-step signaling cascade. Following TLR4 activation, the adaptor protein TIRAP facilitates the recruitment of MyD88 to the intracellular TIR domain of TLR4 (Wang Y. et al., 2020), prompting MyD88 to recruit IRAK4 and trigger its own phosphorylation. Subsequently, IRAK4 mediates the phosphorylation of IRAK1. Highly phosphorylated IRAK1 dissociates from MyD88 and recruits TRAF6 to form a complex (Giménez et al., 2020; Strickson et al., 2017). This complex further interacts with the TAK1-TAB1-TAB2 complex on the cell membrane, promoting the phosphorylation of TAB1 and TAB2. Ultimately, the TRAF6-TAK1-TAB1-TAB2 complex forms and enters the cytoplasm (Zhang J. et al., 2017). In parallel, TLR4 can also recruit the adaptor protein TRAM, leading to activation of the TRIF-dependent pathway involving RIP1, TBK1, IRF3, and delayed NF-κB signaling. However, direct evidence linking this pathway to ferroptosis remains limited. Within the MyD88-dependent pathway, TAK1 functions as a key downstream kinase following IRAK1/4 and TRAF6 activation. Activation of this signaling cascade has been reported to regulate a series of ferroptosis-related targets, thereby influencing the expression and activity of key ferroptosis-associated proteins: MyD88-dependent signaling has been reported to modulate the expression of several ferroptosis-related molecules, including FTH1, HO-1, FPN, and CHAC1. In contrast, TAK1-associated signaling has been implicated in regulating GLS2, FDXR, ACSL4, ALOX15, and NCOA4 expression, while affecting SLC7A11 expression and CAT activity. It also promotes mitoferrin-2 (MFRN2), TFR1, and ALOX12 activation while altering DPP4 subcellular localization, collectively participating in ferroptosis regulation.

**FIGURE 2:**
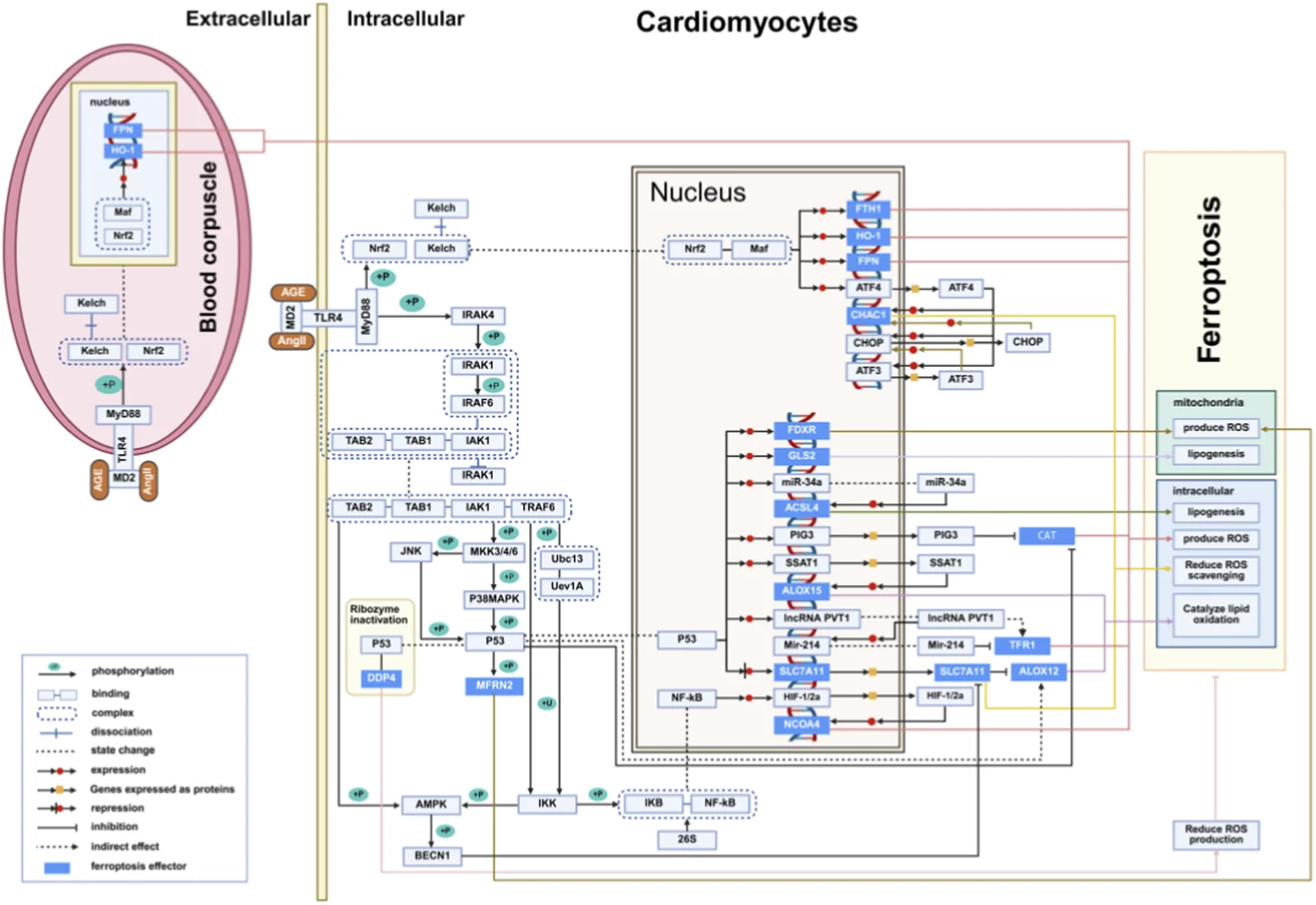
TLR4 signaling regulates ferroptosis in cardiomyocytes. Created in BioRender. Huang, X. (2026) https://BioRender.com/6s4h6jc.

### 3.1 Activation of the TLR4 signaling pathway during heart failure

TLR4 belongs to the pattern recognition receptor family, composed of a 608-residue extracellular domain and an 187-residue intracellular domain, serving as a core component of the mammalian innate immune system. Its primary function is to recognize exogenous molecules from pathogens, particularly lipopolysaccharides. Extensive research indicates that TLR4 also recognizes endogenous molecules released by damaged tissue cells. TLR4 signaling is activated during heart failure progression, and accumulating evidence suggests that endogenous molecules such as advanced glycation end products (AGEs) and angiotensin II (Ang II) may serve as its upstream triggers (Chen et al., 2022).

AGEs originate from high-fat and high-sugar diets and can also be synthesized endogenously. Endogenous formation primarily occurs through the Maillard reaction, in which aldehyde or ketone groups of reducing sugars react with the reductive amino groups of proteins to form Schiff bases, which then undergo structural rearrangement into more stable Amadori early-stage products. These Amadori products can further generate AGEs through multiple pathways, such as the condensation of two Amadori molecules, the reaction between Amadori and 3-deoxyglucosone to form imidazole- or pyrrole-based protein cross-links, or via slow and complex processes involving dehydrogenation, oxidation, and rearrangement (Shen et al., 2020). The formation of AGEs is irreversible, and their stable structure makes them resistant to enzymatic degradation. Clearance depends mainly on phagocytosis by mononuclear macrophages and renal excretion. Aging accelerates the accumulation of AGEs, which in turn promotes the aging process (Chaudhuri et al., 2018).

Excessive activation of the renin-angiotensin system during heart failure progression generates substantial amounts of Ang II. When cardiac function is impaired, renal perfusion volume and pressure fall far below normal levels, consequently reducing stretch stimulation of afferent arterioles in the glomeruli. This triggers compensatory increases in chemical factor release, stimulating excessive activation of sympathetic or vagal afferent nerves that transmit excitation to the central nervous system. Regions such as the solitary tract nucleus and paraventricular nucleus of the hypothalamus release large amounts of catecholamines and cholinergic neurotransmitters. These bind to β-adrenergic receptors in cells surrounding the glomeruli, inducing renin secretion. Renin enters the bloodstream via the renal vein, where it catalyzes the conversion of angiotensinogen released by the liver into angiotensin I. These findings suggest that TLR4 signaling may participate in ferroptosis regulation through downstream transcriptional and post-transcriptional mechanisms, although the specific contribution to HF remains to be further clarified (Paz et al., 2020).

Ligand-induced TLR4 activation requires physical association with MD2 on the cell surface. AGEs accumulated during HF and elevated Ang II levels have been reported to bind to MD2-TLR4 complexes on the cell membrane, thereby promoting TLR4 activation. This signaling pathway has been suggested to contribute to ferroptosis-associated responses (Li et al., 2019). Beyond enhancing the transcriptional levels of inflammatory mediators, TLR4 signaling has been suggested to regulate the transcriptional expression of ferroptosis-related genes and protein activation through intracellular signaling cascades. This influences specific cellular biological effects, thereby exerting a certain impact on the onset and progression of related diseases.

### 3.2 TLR4 signaling regulates ferroptosis proteins

#### 3.2.1 MyD88 regulation of ferroptosis proteins

MyD88-dependent TLR4 signaling has been reported to influence the expression and activation of the transcription factor NRF2 (Kim et al., 2011). Phosphorylated Nrf2 dissociates from Kelch-like ECH-associated protein 1 (KEAP1) and enters the nucleus, where it forms heterodimers with Maf via its Nehl domain. These dimers then bind to antioxidant response element-like binding sites on genes such as FTH1, HO-1, FPN, and ATF4 to initiate transcription (Zhao et al., 2021; DeNicola et al., 2015). Subsequently, ATF4 acts as a transcription factor to recognize and bind the CRE, ATF/CRE modulator element, and ATF/CEBP response element on the CHAC1 gene promoter, thereby promoting CHAC1 expression (Crawford et al., 2015). ATF4 can also bind amino acid regulatory elements to increase CHOP transcription or bind ATF binding sites on the ATF3 promoter to induce ATF3 transcription, thereby promoting CHOP expression (Lee et al., 2013; Tian et al., 2020). This CHOP-mediated transcription of the CHAC1 gene further contributes to the pathway (Wang et al., 2022). Notably, NRF2-regulated molecules, including FTH1, HO-1, and FPN, exert context-dependent effects on ferroptosis. Under physiological conditions, NRF2 activation generally enhances antioxidant defense and iron homeostasis. However, excessive or sustained activation of specific downstream targets, particularly HO-1, may contribute to iron overload and ferroptosis in certain pathological settings. Within the MyD88-dependent pathway, TAK1 functions as a key downstream kinase following IRAK1/4 and TRAF6 activation. Activation of this signaling cascade has been reported to regulate a series of ferroptosis-related targets, thereby influencing the expression and activity of key ferroptosis-associated proteins.

#### 3.2.2 TAK1 regulation of ferroptosis proteins

A recent comprehensive review has systematically delineated this RAGE/TLR4-JNK1/2-ferritinophagy/clockophagy/p53 axis in the context of cardiomyocyte ferroptosis, providing an integrated framework for understanding the crosstalk between TLR4 signaling and ferroptosis in heart failure (Zhang et al., 2025). TAK1 has been reported to phosphorylate and activate MKK4/7 in macrophages (Zhang et al., 2021). In other cellular contexts, the activation of MKK3, MKK4, and p38 MAPK has been implicated in p53-mediated signaling (Guo et al., 2022; He et al., 2020). JNK has been shown to phosphorylate p53 at threonine 81 (Thr81), promoting p53-p73 complex formation and apoptosis. Upstream of JNK, TRAF6 and TAK1 have been implicated in JNK activation (Wang et al., 2015; Wolf et al., 2018). Phosphorylated and activated p53 further regulates the expression of multiple downstream target genes, including regulation of the transcription of GLS2 (Hu et al., 2010), FDXR (Zhang Y. et al., 2017), miR-34a (Shi et al., 2020), SSAT1 (Liang et al., 2021), PIG3 (Kang et al., 2013) and LncRNA PVT1 (Olivero et al., 2020), while simultaneously suppressing SLC7A11 expression (Ma et al., 2020). Additionally, p53 activates MFRN2 (Lill and Freibert, 2020),and sequesters DPP4 in the ribosomal inactivation pool, thereby inhibiting its function (Xie et al., 2017). Among these p53-regulated downstream factors, multiple molecules further participate in regulating related signaling networks: SSAT1 induces ALOX15 overexpression (Zhao et al., 2022); both PIG3 and p53 suppress CAT activity (Kang et al., 2013); LncRNA PVT1 activates TFR1 by inhibiting miR-214 expression (Lu et al., 2020); and downregulation of SLC7A11 indirectly activates ALOX12 (Chu et al., 2019).

Additionally, TAK1 can also target and activate IKK (Antonia and Baldwin, 2018), while TRAF6 acts as an E3 ubiquitin ligase and binds to the ubiquitin-conjugating enzyme Ubc13/Uev1A to form polyubiquitin chains for IKK ubiquitination. Both IKK and TAK1 have been reported to influence AMPK phosphorylation at Thr172 (Antonia and Baldwin, 2018; Neuman and n, 2018). AMPK mediates the phosphorylation of BECN1 at Ser90/93/96, promoting BECN1 binding and inhibiting SLC7A11 activity (Song et al., 2018). Concurrently, IKK phosphorylates IκB; activated IκB is recognized by E3, ubiquitinated, and degraded by the 26S proteasome, exposing the Rel homodomain of NF-κB (Antonia et al., 2021; Cohen and Strickson, 2017). Subsequently, NF-κB binds to the HIF-1/2α promoter and enhances its transcription (Lisk et al., 2013). Although reports indicate that NCOA4 mRNA can be degraded by miR-6862–5p regulated by JNK (Fuhrmann et al., 2020), NCOA4 expression is generally HIF-1/2α-dependent and upregulated (Li et al., 2020).

It should be noted that the mechanistic evidence summarized in the following sections is derived from diverse cell types and disease models. Unless otherwise indicated, many of these findings should be regarded as indirect evidence, and their relevance to cardiomyocytes and heart failure requires further experimental validation.

### 3.3 Ferroptosis proteins and myocardial cell ferroptosis

Ferroptosis differs distinctly from other known forms of cell death in terms of cellular morphology, genetics, and biochemistry. Its primary biochemical feature is the iron-dependent accumulation of lipid peroxides (Dixon and Olzmann, 2024). TLR4-associated ferroptosis-related proteins may exert bidirectional effects on ferroptosis regulation in cardiomyocytes. Within the synergistic regulatory mechanism of ferroptosis in cardiomyocytes, multiple molecular changes collectively promote oxidative stress and lipid peroxidation processes. On one hand, disruption of iron homeostasis and excessive ROS generation are key contributing factors. Although HO-1 and FPN are generally involved in maintaining iron homeostasis, excessive HO-1 activation and altered systemic iron redistribution under pathological conditions may contribute to intracellular iron accumulation and oxidative stress. This may enhance Fenton chemistry and contribute to ROS generation. Although increased FTH1 expression generally promotes iron sequestration and limits the labile iron pool, enhanced NCOA4-mediated ferritinophagy can release stored iron from ferritin, thereby increasing cytoplasmic Fe^2+^ levels and exacerbating ROS generation under specific pathological conditions. A similar mechanism occurs in mitochondria: Studies have suggested that increased FDXR activity may influence respiratory chain function, whereas MFRN2 upregulation may contribute to mitochondrial iron accumulation and oxidative stress. On the other hand, lipid metabolism reprogramming and weakened antioxidant systems jointly amplify the damaging effects: ACSL4 overexpression promotes cell membrane lipid synthesis, while ALOX15 overexpression and ALOX12 activation accelerate lipid peroxidation reactions. Simultaneously, downregulation of SLC7A11 and overexpression of CHAC1 cause GSH depletion, impairing the cell’s ability to clear lipid peroxides. Furthermore, GLS2 upregulation may alter mitochondrial metabolism and redox homeostasis, thereby influencing ferroptosis susceptibility. Conversely, altered subcellular localization of DPP4 in cardiomyocytes reduces ROS generation and inhibits cellular lipid peroxidation. It should be noted that many of the mechanistic links summarized above have been identified in non-cardiac cell types or disease models. Their relevance to cardiomyocyte ferroptosis remains indirect and requires further experimental validation in heart failure models.

#### 3.3.1 HO-1,CAT,FPN,TFR1

HO-1 exhibits context-dependent effects in ferroptosis. Under physiological conditions, HO-1 contributes to heme degradation and protection against oxidative stress, whereas excessive or sustained HO-1 activation may contribute to increased intracellular iron availability and ferroptosis susceptibility under specific pathological conditions. HO-1 degrades heme to produce carbon monoxide, biliverdin, and Fe^2+^, while CAT catalyzes the decomposition of hydrogen peroxide into oxygen and water. The myocardium contains abundant heme for synthesizing myoglobin, cytochromes, etc. In certain pathological contexts, increased iron availability associated with HO-1 activation and impaired hydrogen peroxide clearance due to reduced CAT activity may enhance Fenton/Haber-Weiss chemistry, thereby increasing oxidative stress. This leads to lipid peroxidation of polyunsaturated fatty acids constituting biological membranes, representing a central biochemical process associated with ferroptosis. Fe^2+^ also acts as a cofactor to enhance the activity of various metabolic enzymes, including lipoxygenases and mitochondrial pyruvate dehydrogenase 1, potentially contributing to lipid oxidation during ferroptosis (Fang et al., 2019; Jiang et al., 2021; Abbate and Hider, 2017; Torti and Torti, 2013). FPN generally functions as the only known cellular iron exporter and is therefore considered protective against ferroptosis by facilitating iron efflux. However, under systemic pathological conditions, iron exported through FPN may subsequently become available for uptake by cardiomyocytes via transferrin-TFR1-dependent pathways, potentially contributing to iron redistribution rather than directly promoting ferroptosis. After binding to TFR1 on the cardiomyocyte membrane, the transferrin–TFR1 complex undergoes endocytosis, where Fe^3+^ is reduced to Fe^2+^ by STEAP3 within the endosome before being transported into the cytoplasm via DMT1. Following uptake through TFR1-dependent pathways, intracellular iron may contribute to expansion of the labile iron pool and increased ferroptosis susceptibility under pathological conditions (Park and Chung, 2019; Menon et al., 2022; Gao et al., 2015).

#### 3.3.2 FTH1,NCOA4

FTH1 possesses ferroxidase activity, oxidizing unused or expelled Fe^2+^ in the cytoplasm to Fe^3+^ for storage in ferritin (Plays et al., 2021). Although studies indicate Fe^3+^ can be expelled through ferritin pores, the key mechanism for Fe^3+^ release is NCOA4-mediated ferritinophagy (Radisky and Kaplan, 1998; Ito et al., 2021). NCOA4 binds to the FTH1 subunit of ferritin via a conserved C-terminal domain and interacts with the light chain 3-phosphatidylethanolamine of microtubule-associated protein 1 on developing autophagosome membranes. This complex sequesters Fe^3+^-containing ferritin within autophagosomes. Upon autophagosome maturation and fusion with lysosomes, Fe^3+^ is released during ferritin degradation where it is subsequently reduced back to Fe^2+^ by FDX2 (Zhang Y. et al., 2017; Kaur and Debnath, 2015). Increased FTH1 transcription generally enhances iron storage within ferritin and limits labile iron availability. However, when ferritinophagy is concurrently activated, NCOA4-mediated ferritin degradation releases stored iron, resulting in localized Fe^2+^ accumulation that promotes ferroptosis. Excess Fe^2+^ promotes radical formation through Fenton/Haber-Weiss reactions with hydrogen peroxide, enhances activity of multiple metabolic enzymes, accelerates cellular lipid oxidation, and induces cellular toxicity, potentially contributing to cardiomyocyte injury and functional impairment. Importantly, FTH1 itself is generally regarded as an anti-ferroptotic protein because it sequesters excess iron within ferritin. Increased labile iron is mainly associated with NCOA4-mediated ferritinophagy, which releases stored iron from ferritin. Therefore, the overall effect of FTH1 on ferroptosis depends on the balance between iron storage and ferritin degradation. However, research on ferritin’s role in ferroptosis remains largely confined to *in vitro* cell culture systems. Its physiological and pathophysiological regulation of ferroptosis *in vivo* warrants further investigation.

#### 3.3.3 ACSL4

Lipidomics studies indicate that phosphatidylethanolamine (PE), rich in AA and its derivative DA, is a key phospholipid species associated with ferroptosis (Doll et al., 2017). The activation of long-chain free fatty acids is a prerequisite for their cellular utilization. Upon ACSL4 activation, it catalyzes the synthesis of AA-CoA/DA-CoA from free AA/DA in concert with CoA and ATP (Kuwata and Hara, 2019; Yuan et al., 2016). These intermediates are transported to the cell membrane via lysophosphatidylcholine acyltransferase 3, where esterification generates highly oxidizable membrane components such as AA-PE/ADA-PE (Shimbara-Matsubayashi et al., 2019). Lipid peroxides activate their sensor molecule—PKCβII—which phosphorylates ACSL4 at position T328, promoting ACSL4 activation (Zhang et al., 2022). In certain models, an ACSL4-lipid peroxidation-PKCβII-ACSL4 positive feedback loop has been proposed to amplify lipid peroxidation. This reduces cell membrane fluidity and permeability, increasing ferroptosis susceptibility in cardiomyocytes.

#### 3.3.4 ALOX12/15

ALOX12 and ALOX15, as lipid oxygenation enzymes, jointly participate in redox reactions within cardiomyocytes. ALOX12 primarily catalyzes the metabolism of arachidonic acid (AA) to produce 12-peroxy-eicosapentaenoic acid (12-HPETE) (Mitsui et al., 2021). In contrast, ALOX15 exhibits broader substrate specificity, acting on various free polyunsaturated fatty acids (including AA, linoleic acid, and docosahexaenoic acid), membrane phospholipids (such as AA-PE and ADA-PE), and lipoproteins. In metabolic pathways, AA can be catalyzed by ALOX15 to form 15(S)-HPETE, which can then be reduced by GPX4 to 15(S)-HETE; Both metabolites activate leukotriene B4 receptor 2 (BLT2) and peroxisome proliferator-activated receptor gamma (PPARγ), and at elevated concentrations induce ROS generation. Additionally, ALOX15 catalyzes the combination of linoleic acid with oxygen to form hydrogen peroxyoctadecadienoic acid (HpODE), converts docosahexaenoic acid to 17(S)-hydroxy-docosahexaenoic acid, and oxidizes AA-PE and ADA-PE into their respective phospholipid hydroperoxides, PE-AA-OO(O) and ADA-OO(O), respectively (Singh and Rao, 2019; Ivanov et al., 2015). When abnormal activation of ALOX12 coexists with overexpression of ALOX15, both enzymes accelerate the oxidation of these polyunsaturated fatty acids, disrupting myocardial lipid homeostasis and potentially contributing to ferroptotic responses and myocardial injury.

#### 3.3.5 SLC7A11,CHAC1

The catalytic subunit SLC7A11 and the chaperone subunit solute carrier family 3 member 2 (SLC3A2) form the system xc^−^ cystine/glutamate antiporter through a disulfide bond in the plasma membrane. Inhibition of SLC7A11 transcription and activity restricts the 1:1 exchange of intracellular glutamate and extracellular cystine, thereby limiting the intracellular reduction of cystine to cysteine, a NADPH-dependent process required for GSH synthesis. CHAC1 promotes GSH degradation into cysteinylglycine and 5-oxoproline, thereby reducing intracellular GSH availability (Kumar et al., 2012). GPX4 specifically and efficiently catalyzes the conversion of phospholipid hydroperoxides into phospholipid alcohols, maintaining phospholipid bilayer homeostasis and thereby suppressing ferroptosis (Tsubouchi et al., 2019). GSH serves as the reducing substrate for GPX4. GSH depletion impairs the GSH-dependent reduction of the selenocysteine active site in GPX4, leading to loss of GPX4 enzymatic activity and impaired antioxidant capacity, which lacks reducing activity and weakens cellular antioxidant capacity. Phospholipid hydroperoxides such as malondialdehyde and 4-hydroxy-2-nonenal cannot be cleared promptly, promoting cross-linking and polymerization of biomacromolecules like proteins and nucleic acids, thereby increasing lipid peroxide accumulation and ferroptosis susceptibility in cardiomyocytes (Ursini and Maiorino, 2020).

#### 3.3.6 FDXR

FDXR is a mitochondrial flavoprotein involved in electron transfer from NADPH to ferredoxin proteins, including FDX2, cytochrome P450 reductase, cytochrome b5, and cytochrome P450 (Becker et al., 2018). Within this system, FDXR first accepts electrons from NADPH and transfers them to FDX2 (Chikuma et al., 2021). Subsequently, FDX2 utilizes its iron-sulfur cluster to further transfer electrons to the flavoprotein domain of cytochrome P450. Cytochrome P450 utilizes these electrons to catalyze the hydroxylation of substrates such as arachidonic acid, estradiol, and arachidonic acid via monooxygenation under normal oxygen conditions. Under hypoxic conditions, this enzyme can also utilize peroxides as an oxygen source to complete the hydroxylation of lipid substrates. Furthermore, cytochrome P450 catalyzes the generation of superoxide and peroxides through redox mechanisms, thereby participating in the oxidation of lipid molecules (Guengerich, 2019; Manikandan and Nagini, 2018). In cardiomyocytes, the sustained progression of these electron transfer and oxidative reactions leads to substantial accumulation of lipid peroxides within mitochondria. This accumulation triggers mitochondrial crimping, increased membrane density, and structural and functional disruption, potentially contributing to mitochondrial abnormalities associated with ferroptosis.

#### 3.3.7 GLS2

GLS2 catalyzes the conversion of glutamine to glutamate, which to some extent promotes the production of GSH and reduced coenzyme I to limit ROS. However, studies confirm that glutamine metabolism has been implicated in ferroptosis induced by cysteine deficiency (Johnson et al., 2018). Glutamate is converted by transaminases into α-ketoglutarate for ATP production in the mitochondrial tricarboxylic acid cycle (Altman et al., 2016). During cellular energy metabolism, increased ATP synthesis reduces the AMP/ATP ratio, thereby inhibiting mitochondrial AMPK phosphorylation. Decreased AMPK activity upregulates its downstream substrate, acetyl-CoA carboxylase 1. This enzyme utilizes ATP to fix carbon dioxide onto its cofactor biotin, transferring it to acetyl-CoA to form malonyl-CoA. Subsequently, under the catalysis of fatty acid synthase, malonyl-CoA condenses with acetyl-CoA to form polyunsaturated fatty acids. Concurrently, during electron transfer from the mitochondrial electron transport chain to oxygen molecules, some oxygen molecules undergo incomplete reduction, generating reactive oxygen species such as superoxide anion and hydrogen peroxide (Herzig and Shaw, 2018; Garcia and Shaw, 2017; Gao et al., 2019). Intense peroxidation reactions occur between abundant polyunsaturated fatty acids and ROS within myocardial cell mitochondria. Together with cysteine depletion, these metabolic alterations may increase susceptibility to mitochondrial ferroptosis.

#### 3.3.8 MFRN2

MFRN2 is localized to the inner mitochondrial membrane and primarily mediates the transport of Fe^2+^ from the cytoplasm into the mitochondrial matrix (Hung et al., 2013). Its activation enhances mitochondrial uptake of Fe^2+^, with a portion subsequently stored in mitochondrial ferritin (Maccarinelli et al., 2014; Wu et al., 2016), and another portion participating in the synthesis of heme and iron-sulfur clusters. Iron-sulfur clusters are not only essential components of mitochondrial electron transport chain complexes I, II, and III but also serve as cofactors for succinate synthase and succinate dehydrogenase in the tricarboxylic acid cycle (Lill and Freibert, 2020). Altered mitochondrial iron availability may influence electron transport chain activity and ROS production (Cell Biology of the Mitochondrion, 2018). Furthermore, Fe^2+^ can directly catalyze ROS generation via the Fenton reaction or Haber-Weiss reaction. The accumulation of large quantities of ROS within mitochondria attacks the lipid system, triggering oxidative damage that ultimately leads to mitochondrial dysfunction and promotes ferroptosis in cardiomyocytes. However, current evidence linking TLR4 signaling directly to MFRN2-dependent mitochondrial iron uptake is still limited, and this mechanism has largely been inferred from experimental studies in non-cardiac systems.

#### 3.3.9 DPP4

DPP4 has been reported to form a DPP4-NOX1 complex with NOX1 and undergo endocytic trafficking (Xie et al., 2017). Subsequently, NOX1 utilizes two heme molecules to transfer an electron from NADPH on the cytoplasmic side of the phagosome membrane to an oxygen molecule on the lumen side, promoting the generation of superoxide anions within the phagosome lumen (Salazar, 2018; Parascandolo and Laukkanen, 2019). It has been proposed that sequestration of DPP4 into the nucleolar inactivation pool may cause dissociation of the DPP4-NOX1 complex, thereby reducing superoxide production and potentially limiting lipid peroxidation and ferroptotic responses. However, research on the relationship between NOX1 and cardiomyocyte ferroptosis remains insufficient and warrants further investigation (Table 1).

**TABLE 1:** Summary of experimental evidence supporting the association between TLR4 signaling and ferroptosis in cardiac and non-cardiac models.

Cardiac evidence	Species/Cell type	Model	Key molecule/Pathway	TLR4 investigation	Ferroptosis identification	Rescue experiment	Cardiac/HF relevance	Author and year
Direct	Sprague-Dawley rats; rat H9C2 cardiomyocytes	rat MI/R model; H9C2 hypoxia/reoxygenation (HR) model	USP22; SIRT1; p53 deacetylation; SLC7A11↑; GSH↑; lipid ROS↓; iron accumulation↓; ferroptosis inhibition	No	GPX4; SLC7A11; GSH; lipid ROS; iron	Yes (Fer-1)	Direct	Ma et al. (2020)
	Townes SCD mice: HbSS sickling mice; HbAS littermate controls	SCD mouse mode	Free heme↑; Hmox1↑; iron release↑; cardiac iron overload; lipid peroxidation (MDA)↑; Ptgs2↑; ferroptosis; cardiomyopathy	No	MDA; Ptgs2; iron overload	Yes (Fer-1; DFO)	Direct	Menon et al. (2022)
	Ncoa4 KO mice; C57BL/6J mice; adult mouse cardiomyocytes	TAC pressure overload model	NCOA4; ferritinophagy; FTH1; Fe^2+^; lipid ROS; MDA; 4-HNE; Ptgs2; ferroptosis	No	MDA; 4-HNE; lipid ROS; Ptgs2; iron; mitochondrial morphology	Yes (Fer-1)	Direct	Ito et al. (2021)
	Human cancer lines; mouse B16/4T1; neonatal rat cardiomyocytes; C57/BALB/c mice	Erastin/RSL3-induced ferroptosis model; PD1 immunotherapy model; cardiomyocyte I/R model	PKCβII; ACSL4 Thr328 phosphorylation; PUFA-PE; lipid peroxidation; ferroptosis amplification	No	C11-BODIPY; MDA; ACSL4 phosphorylation	Yes (Fer-1; Lipro-1)	Indirect	Zhang et al. (2022)
	MEFs (WT, RIP3^−/−^, bax/bak DKO); isolated mouse heart	Amino acid/cystine starvation model; *ex vivo* heart I/R model	Transferrin; TfR; glutamine; GLS2; glutaminolysis; GSH depletion; ROS; ferroptosis; compound 968	No	GSH; ROS; iron; lipid peroxidation	Yes (Fer-1; DFO; compound 968)	Direct	Gao et al. (2015)
	C57BL/6J mice; mouse BMDMs; H9c2 cells	CLP-induced septic cardiomyopathy model	Ang II/AT1R; TLR4/NF-κB/MAPK; macrophage polarization; oxidative stress; mitochondrial dysfunction	Direct (TLR4/MyD88)	No ferroptosis markers	No	Direct	Chen et al. (2022)
	hiPSC-CMs	X-ray irradiation (5 Gy, 48 h); RNA-seq transcriptomics	FDXR↑; SPATA18↑; oxidative stress; mitoc hondrial dysfunction; calcium homeostasis	No	No ferroptosis markers	No	Direct	Becker et al. (2018)
	FtMt KO mice	Doxorubicin-induced cardiotoxicity model	FtMt; mitochondrial iron; oxidative damage; TBARS; HO-1; mitochondrial cristae loss	No	TBARS; mitochondrial iron	No	Direct	Maccarinelli et al. (2014)
	FtMt KO mice	Exhaustive exercise-induced myocardial injury model	FtMt; mitochondrial iron accumulation; oxidative stress; MDA; SOD; mitochondrial abnormalities	No	MDA; mitochondrial iron	No	Direct	Wu et al. (2016)
Indirect	MEFs (WT, RIP3^−/−^, bax/bak DKO); isolated mouse heart	Erastin-induced ferroptosis model; xenograft tumor model	p53; DPP4; p53-DPP4 complex; NOX1; lipid peroxidation; SLC7A11; GSH; MDA; ferroptosis	No	MDA; C11-BODIPY; GSH; SLC7A11	Yes (Fer-1; Lipro-1; DPP4 inhibitors)	Indirect	Xie et al. (2017)
	C57BL/6 mice; mouse primary cortical neurons	tMCAO/R model; TBH-induced oxidative stress model; SSAT1 knockdown/overexpression models	SSAT1; ALOX15; GPX4; SLC7A11; lipid peroxidation; iron; ROS; ferroptosis	No	GPX4; SLC7A11; MDA; 4-HNE; iron; ROS	Yes (Fer-1; PD146176)	Indirect (cerebral I/R)	Zhao et al. (2022)
	C57BL/6 mice; PC12 cells; clinical AIS plasma samples	tMCAO/R model; OGD/R PC12 model	lncRNA PVT1; miR-214; TFR1; p53; SLC7A11; GPX4; GSH; lipid peroxidation; iron; ferroptosis	No	GPX4; SLC7A11; TFR1; iron; MDA; GSH/GSSG	Yes (Fer-1)	Indirect (cerebral I/R)	Lu et al. (2020)
	Human cancer lines; MEFs (WT, Alox12^+/−^, Alox12^−/−^, Acsl4^−/−^); Eμ-Myc transgenic mice; nude mouse xenograft	p53-mediated ferroptosis model; erastin/RSL-3-induced ferroptosis model; xenograft tumor model	p53; SLC7A11; ALOX12; lipid peroxidation; PUFA oxidation; ferroptosis (ACSL4-independent); p53-ALOX12-SLC7A11 axis	No	ALOX12 activity; lipid peroxidation; Ptgs2; CHAC1	Yes (Fer-1; ML-355)	Indirect	Chu et al. (2019)
	Human cancer lines; mouse PDAC KPC cells; nude mouse xenograft; KPC orthotopic model	System xc^−^ inhibitor-induced ferroptosis model; xenograft/orthotopic tumor model	AMPK; BECN1 phosphorylation (Ser90/93/96); BECN1-SLC7A11 complex; system xc^−^ inhibition; GSH depletion; lipid peroxidation; ferroptosis	No	MDA; C11-BODIPY; GSH; Fe^2+^	Yes (Fer-1; Lipro-1)	Indirect	Song et al. (2018)
	Primary human macrophages; HT1080 cellsPrimary human macrophages; HT1080 cells	Hypoxia model; RSL-3-induced ferroptosis modelHypoxia model; RSL-3-induced ferroptosis model	JNK; miR-6862–5p; NCOA4; ferritinophagy; FTMT; FTH; iron storage; resistance to ferroptosis	No	FTMT; FTH; NCOA4; labile iron pool	Yes (Lipro-1)	Indirect	Fuhrmann et al. (2020)
	Mouse embryonic fibroblasts (WT, BECN1^+/−^, LC3B^−^/^-^); human primary fibroblasts	Erastin-induced ferroptosis model	Erastin; ROS; autophagy; BECN1; LC3B; ferritin degradation; TfR1; iron; lipid peroxidation; ferroptosis	No	Iron; lipid peroxidation (C11-BODIPY); ROS	No	Indirect	Park and Chung (2019)
	Fdxr KO mice; MEFs; human HCT116; H1299 cells	RSL3/erastin-induced ferroptosis model	FDXR; FDX2; IRP2; TfR1; FTH1; mitochondrial iron; Fe-S cluster; p53; ferroptosis	No	Mitochondrial iron overload; TfR1; FTH1; IRP2	No	Indirect	Zhang et al. (2017)
	Mouse RAW264.7 macrophages; mouse BMDM	LPS-induced inflammation model	TLR4; MyD88; Nrf2; NQO-1; HO-1; GCLC; ROS-independent Nrf2 activation	Direct (TLR4/MyD88)	No ferroptosis markers	No	Indirect	Kim et al. (2011)
​	Human HepG2 cells	Alcohol-induced ferroptosis model	Autophagy inhibition; p62; Keap1; Nrf2; FTH; FPN; HO-1; iron storage/efflux; antioxidant defense; ferroptosis inhibition	No	GPX4; ACSL4; iron; MDA; GSH; lipid ROS	Yes (Fer-1)	Indirect	Zhao et al. (2021)
	Human NSCLC lines; nude mouse xenograft	Serine/glycine starvation model; xenograft tumor model	NRF2; ATF4; PHGDH; PSAT1; SHMT2; serine/glycine synthesis; GSH; NADPH; nucleotide synthesis	No	No ferroptosis markers	No	Indirect	DeNicola et al. (2015)
	HEK293; U2OS cells	ER stress model; amino acid starvation model	ATF4; ATF3; CEBPβ; CHAC1; GSH degradation; GSH depletion; ER stress; oxidative stress; apoptosis	No	CHAC1; GSH degradation	No	Indirect	Crawford et al. (2015)
	Human CRC cells (HCT-116, SW480, HT-29, LoVo, Caco-2)	DIM treatment model	DIM; ATF4; ATF3; apoptosis; ER stress; GSH depletion	No	No ferroptosis markers	No	Indirect	Lee et al. (2013)
	Human lens epithelial cells (HLECs)	UVB radiation-induced oxidative stress model	KLF6; ATF4; ATF3; CHOP; ER stress; apoptosis; ROS; Bax/Bcl-2	No	No ferroptosis markers	No	Indirect	Tian et al. (2020)
	Human OSCC lines; mouse SCC7; BALB/c nude mouse xenograft	GLA treatment model; xenograft tumor model	GLA; ROS; ATF4; CHOP; CHAC1; GSH degradation; ER stress; mitochondrial apoptosis; 4-HNE	No	CHAC1; GSH; 4-HNE	No	Indirect	Wang et al. (2022)
	Mouse RAW264.7; HEK293T cells	LPS-induced inflammation model	TAK1; MKK4/7; MEK1/2; JNK; p38; ERK; AP-1; iNOS; COX-2; IL-1β; IL-6; NO	No	No ferroptosis markers	No	Indirect	Zhang et al. (2021)
	Mouse B16-F10; human A375; BALB/c nude mouse xenograft	RIG-I KO/OE model; xenograft tumor model	RIG-I; MKK3; MKK4; p38 MAPK; cell cycle arrest; Cyclin D1; apoptosis	No	No ferroptosis markers	No	Indirect	Guo et al. (2022)
	C57BL/6 mice (aging); Villin-Cre; Tsc1f/f; Lgr5-CreERT; Tsc1f/f; Trp53^−/−^/Cdkn2a−/− mice; intestinal organoids	Tsc1 ablation-induced aging model	mTORC1; MKK6; p38 MAPK; p53; p16; ISC/progenitor proliferation; crypt/villus aging	No	No ferroptosis markers	No	Indirect	He et al. (2020)
	Human cancer lines (MDA-MB-468, TMD231, MDA-MB-157, H1299, U87, SF767); BJ fibroblasts; mouse 3T3; HME1	Anisomycin/camptothecin-induced apoptosis model	JNK; p53 Thr81 phosphorylation; p53-p73 complex; PUMA; Bax; apoptosis; mutant p53 dominant-negative	No	No ferroptosis markers	No	Indirect	Wolf et al. (2018)
	Human cancer lines (H1299, HCT116, HepG2, LN-2024, H460, HTB-15); clinical HCC samples	p53 activation model; HCC model	p53; miR-34a; CSF1R; STAT3; SNAIL; EMT; invasion; metastasis; 5-FU resistance	No	GSH; ROS	No	Indirect	Hu W, et al. (2010)
​	Human CRC lines (HCT15, RKO, DLD1, SW480, SW620, HT29); NOD/SCID mouse xenograft	5-FU resistance model; lung metastasis model	p53; miR-34a; CSF1R; STAT3; SNAIL; EMT; invasion; metastasis; 5-FU resistance	No	No ferroptosis markers	No	Indirect	Shi et al. (2020)
	Human trophoblastic cells (Swan 71, HTR-8/SVneo); C57BL/6 pregnant mice; clinical RM/HC villous tissues	BPDE/BaP-induced miscarriage model	Lnc-HZ03; miR-hz03; p53; SAT1; spermine metabolism; trophoblastic cell apoptosis; miscarriage	No	No ferroptosis markers	No	Indirect	Liang et al. (2021)
	Human cancer lines (U2OS, RKO, HCT116, H1299, H460, SW480, Saos-2); HEK293T	UV irradiation model; H_2_O_2_-induced oxidative stress model	p53; PIG3; p53R2; catalase; ROS regulation; p53 dual role (prooxidant vs. antioxidant); apoptosis	No	ROS	No	Indirect	Kang et al. (2013)
	WT MEFs; p53-proficient/deficient MEFs; KPR cells; KC/KPC mouse lung cancer models	Doxorubicin/Tamoxifen-induced p53 activation model; lung cancer model	p53; Pvt1b; Myc transcriptional repression; cell proliferation; tumor growth suppression	No	No ferroptosis markers	No	Indirect	Olivero et al. (2020)
	Recombinant human ACSL4V1/V2 in Sf9 cells; Caco-2 cells	*In vitro* ACSL enzyme assay model	ACSL4V1; ACSL4V2; AA; EPA; DHA; adrenic acid; acyl-CoA synthesis; PUFA-phospholipid synthesis; ferroptosis	No	Enzymatic activity only	No	Indirect	Shimbara-Matsubayashi et al. (2019)
	Recombinant ChaC1/YER163c in *E. coli*; *S. cerevisiae* strains	*In vitro* ChaC enzyme assay model; yeast glutathione degradation model	ChaC1; CHAC1; YER163c/GCG1; γ-glutamyl cyclotransferase; GSH degradation; GSH depletion; apoptosis	No	GSH degradation only	No	Indirect	Kumar et al. (2012)
	Human lung fibroblasts; GPx4^+/−^ and GPx4 transgenic mice; C57BL/6J mice	TGF-β-induced myofibroblast differentiation model; BLM-induced lung fibrosis model	GPx4; lipid peroxidation; 4-HNE; MDA; HODE/HETE; TGF-β; SMAD2/3; myofibroblast differentiation; lung fibrosis; ferroptosis	No	GPX4; 4-HNE; MDA; HODE; HETE	No	Indirect	Tsubouchi et al. (2019)
	Human head and neck squamous carcinoma cell lines: UMSCC1, UMSCC14A, UMSCC22A	PDT model (photodynamic therapy)	Mfrn2; mitochondrial iron uptake; lysosomal iron release; Fenton reaction; ROS; mitochondrial dysfunction; iron-dependent cell death	No	Mitochondrial iron uptake	No	Indirect	Hung et al. (2013)
	Mouse RAW264.7 macrophages; mouse 32D myeloid progenitor cells	GM-CSF stimulation model	GM-CSF; TRAF6; K63-ubiquitination; JNK; p38; Akt; TAK1; cell proliferation; cell survival	No	No ferroptosis markers	No	Indirect	Wang et al. (2015)
	Porcine primary intramuscular preadipocytes; HEK293T cells	Preadipocyte differentiation model	miR-34a; ACSL4; PPARγ; aP2; SREBP-1C; ATGL; Sirt1; lipid droplet formation; adipogenesis; ferroptosis	No	ACSL4 (enzymatic only)	No	Indirect	Wang et al. (2020)

## 4 Therapeutic strategies targeting TLR4-Associated ferroptotic responses

### 4.1 Ferroptosis inhibitors and iron chelators

Pharmacological and natural interventions reported to alleviate TLR4-associated ferroptotic responses can be broadly classified according to their primary mechanisms of action into ferroptosis inhibitors, iron chelators, direct TLR4/MD2 signaling inhibitors, and natural compounds with potential multi-target effects. Ferrostatin-1, a radical-trapping antioxidant, has been reported to alleviate ferroptotic responses in lipopolysaccharide-induced septic myocardial dysfunction models, accompanied by suppression of the TLR4/NF-κB/IκBα inflammatory pathway. This was associated with reduced levels of inflammatory mediators in myocardial tissue and serum, decreased ferroptosis-associated myocardial injury, and improved myocardial contractility (Xiao et al., 2021). Ferrostatin-1 has also been reported to alleviate ferroptotic changes in pulmonary artery endothelial cells in a colchicine-induced pulmonary hypertension model, accompanied by modulation of the HMGB1/TLR4/NLRP3 pathway (Xie et al., 2022).

### 4.2 TLR4/MD2 signaling pathway inhibitors

The specific TLR4 inhibitor TAK-242 suppresses TLR4/p38 MAPK signaling, alleviating neuronal oxidative stress and mitochondrial damage in a neonatal rat model of hypoxic-ischemic brain injury. This intervention was associated with reduced ferroptotic changes and improved learning and memory (Zhu et al., 2021). The MD2 inhibitor L6H21 alleviates myocardial cell mitochondrial dysfunction by blocking MD2/TLR4 complex formation, thereby suppressing oxidative stress and inflammatory responses. This reduces apoptosis and ferroptosis, improves cardiac autonomic function and left ventricular function in prediabetic rats, and exerts cardioprotective effects (Sumneang et al., 2022).

### 4.3 Natural compounds with potential multi-target effects

In addition to these pharmacological agents, several natural compounds have demonstrated potential protective effects against ferroptosis through multiple mechanisms, including modulation of TLR4 signaling. Curcumin has been reported to alleviate ferroptosis-associated cell death in acute kidney injury induced by rhabdomyolysis by inhibiting the TLR4/NF-κB signaling pathway (Guerrero-Hue et al., 2019). Liensinine has been reported to attenuate hypertension-induced vascular remodeling by suppressing ferroptosis-related TLR4 signaling. It disrupts TLR4/MD2 complex formation, inhibits MyD88/NF-κB activation, restores GPX4 expression, and suppresses downstream MAPK and TGF-β1/Smad2/3 signaling, thereby reducing vascular inflammation and remodeling (Chen et al., 2025). Salvianolic acid B, stigmasterol, and resveratrol can bind to TLR4 and inhibit neutrophil extracellular trap formation. Their protective effects against myocardial infarction-induced injury may be related to regulating immune cell infiltration and suppressing ferroptosis (Jiang et al., 2022). The traditional Chinese compound preparation Huazhi Fugan Granules similarly treats ferroptosis-involved non-alcoholic fatty liver disease progression by inhibiting the TLR4/NF-κB pathway (Ye et al., 2021).

Although research on the molecular regulatory mechanisms of TLR4 and its association with ferroptosis in cardiomyocytes is still in its infancy, and relevant inhibitors have yet to be formally launched, experimental studies on TLR4 inhibition therapy in preliminary ferroptosis cell/animal models have achieved a series of significant advances. These findings have charted a course for future research and instilled confidence in the clinical translation of ferroptosis studies. Overall, currently available agents differ substantially in their mechanisms of action. Some directly inhibit TLR4 signaling, whereas others suppress ferroptosis through radical-trapping antioxidant activity or iron chelation, while natural compounds generally exert broader multi-target effects (Figure 3).

**FIGURE 3:**
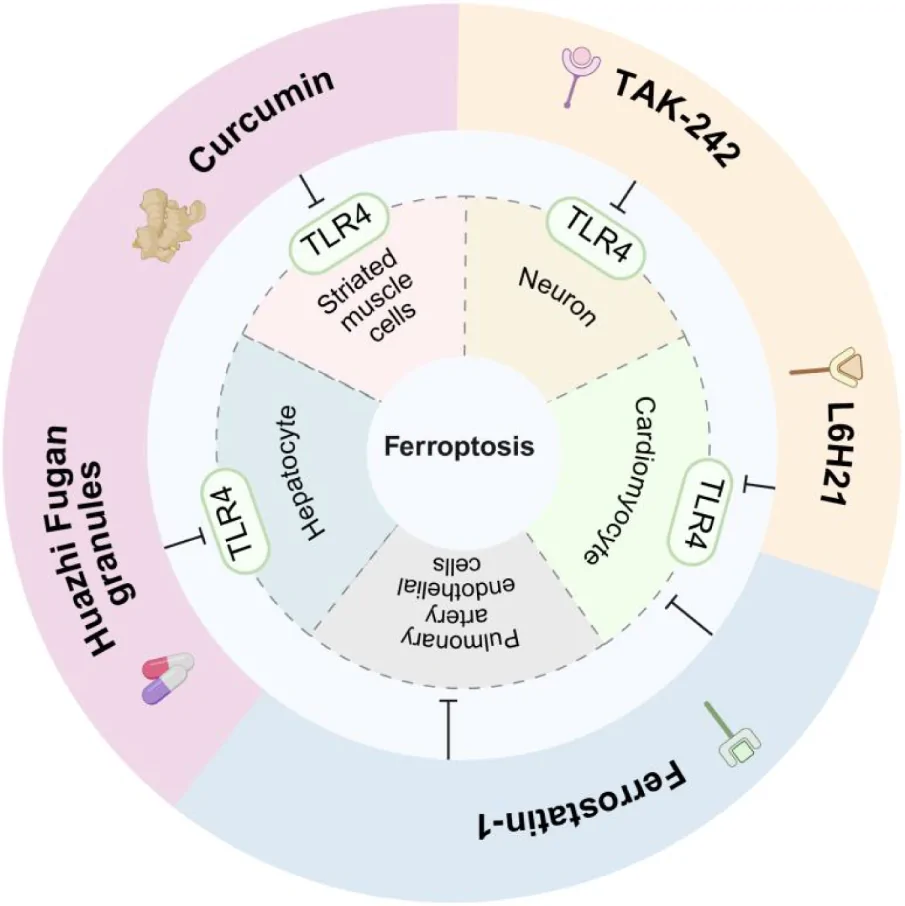
Therapeutic agents reported to alleviate TLR4-associated ferroptosis. TAK-242 and L6H21 directly inhibit TLR4 signaling, whereas Ferrostatin-1 suppresses ferroptosis by scavenging lipid radicals in cardiomyocytes and pulmonary artery endothelial cells. Curcumin and Huazhi Fugan Granules alleviate ferroptosis through multi-target mechanisms involving TLR4-associated signaling in striated muscle cells and hepatocytes, respectively. Created in BioRender. Huang, X. (2026) https://BioRender.com/fid066p.

## 5 Conclusion and perspectives

Cardiomyocyte ferroptosis has been implicated in the progression of HF and may contribute to cardiac structural and functional impairment. Undoubtedly, inhibiting cardiomyocyte ferroptosis is a key strategy for protecting cardiac function and preventing HF. As a classic inflammatory regulatory pathway, the potential involvement of TLR4 signaling in cardiomyocyte ferroptosis has attracted increasing attention as research in this field continues to expand. TLR4 appears to be activated by AGEs and Ang II accumulated during HF progression, exerting dual effects on cardiomyocyte ferroptosis. Current evidence suggests that TLR4 signaling may contribute to ferroptosis through the regulation of multiple ferroptosis-related molecules: it has been reported to regulate multiple ferroptosis-related molecules, including FTH1, HO-1, FPN, CHAC1, GLS2, FDXR, ACSL4, ALOX15, and NCOA4, although the biological effects of NRF2-regulated molecules (particularly FTH1, HO-1, and FPN) remain context-dependent and may vary according to cellular environment and disease stage. These interconnected mechanisms are proposed to facilitate lipid peroxide accumulation and may ultimately contribute to cardiomyocyte ferroptosis. TLR4-associated alteration of DPP4 subcellular localization may lead to dissociation of the DPP4-NOX1 complex, reducing superoxide anion production and inhibiting lipid peroxidation and ferroptosis. However, evidence supporting this remains insufficient and requires further validation.

In summary, the pivotal role of ferroptosis in the progression of major diseases such as HF has garnered widespread attention, with new targets and mechanisms continually emerging. Targeting TLR4-associated ferroptotic pathways may represent a potential therapeutic strategy for the prevention and management of HF. Although accumulating evidence supports a close association between TLR4 signaling and ferroptosis, many mechanistic links remain to be directly validated in cardiomyocytes and *in vivo* models of heart failure. Future studies should also adopt more rigorous criteria for ferroptosis identification, including direct assessment of iron-dependent lipid peroxidation, together with pharmacological or genetic validation of core ferroptosis regulators, to further strengthen the mechanistic evidence linking TLR4 signaling to ferroptosis in heart failure. Further in-depth research is expected to elucidate the intricate molecular mechanisms by which the TLR4 signaling pathway regulates ferroptosis in cardiomyocytes, thereby providing robust scientific evidence for the clinical application of TLR4-targeted therapies to prevent and treat cardiomyocyte ferroptosis. Given this, it is anticipated that with emerging experimental and clinical research findings alongside technological breakthroughs, specific drugs capable of intervening in multiple signaling pathways of TLR4 may be developed in the future. These interventions could prevent ferroptosis in cardiomyocytes and delay the progression of HF.
